# Monthly Summary

**Published:** 1884-07

**Authors:** 


### MONTHLY SUMMARY.

A DENTIST'S BILL.—The following appeared in the New York *Times* of July 2nd, concerning a bill which was sent to Gen. Blanco, the Venezuelan President, last week, for professional work, for Mrs. Blanco and two of her daugthers. The three ladies, acting on the advice of a friend in this city, went to Dr. Atkinson, at No. 41 East Ninth-street, and arranged with him for treatment. This was one week ago last Monday, and as the President and his family were going to sail on the following Saturday, there was necessity for haste. Dr. Atkinson set aside other engagements, hired additional help, and went to work. The day before the family sailed, the ladies left the doctor's office believing that their teeth were as sound as dental skill could make them, and professedly satisfied with the work.

A bill was sent to President Blanco charging $7,000 for the service. He was somewhat astonished at what he believed to be an enormous price for four day's work, and showed the bill to a friend. The friend was likewise astounded. He put the bill in his pocket, took it into the billiard-room, and told the story as a capital joke to his acquaintances. It was repeated over the billiard tables, drifted into the bar-room, stirred up with the social milk punch, and was chuckled at by professional men in the reading-room. President Blanco was not at all pleased with the notoriety he was getting, and would have paid the bill to avoid publicity, but his friends dissuaded him from doing so. The gentlemen to whom he had shown the bill in the first place, called on Dr. Atkinson, and suggested that perhaps there was a mistake.

"Oh, no," said the doctor, "not at all. That is the amount of my bill."

The friends went away protesting that the sum was an imposition and would not be paid. President Blanco went away, and left a sum of money (said to be $1,500) in the hands of a business firm whom he had some dealings with, requesting them to come to an amicable settlement if they could do so.

Dr. Atkinson lost his temper when he heard that other people were inclined to interfere in his business and make the matter public, but he declined to have anything to say for purely professional reasons. A fellow-dentist, who had talked over the matter with Dr. Atkinson, and was acquainted with the facts, said: "Dr. Atkinson considers his professional services worth $50 an hour, and if people go to him I suppose they must expect to pay him his price. Mrs. Blanco knew that her bill whuld be large, though there were a few charges which she probably did not take into consideration. Dr. Atkinson's three sons are engaged with him in the business. When he employs them on his own patients of course he pays them by the hour. I am told that the dentists worked with the three ladies in the chair from early morning until dark, and the artificial part was done on extra time in the evenings. Of course that went into the bill. Then, at President Blanco's request, all other engagements were set aside until this work was done. That involved loss of custom, which was probably taken into consideration."

Dr. Atkinson displays in a frame near his operating-chair engrossed resolutions from the money-order department of the New-York Post Office, thanking him for saving the life of their Superintendent by curing an attack of "necrosis of the jaw, a painful process of decay."

If a compromise is not reached an appeal will be made to the courts.

---

TEXTILE METALLIC FILLING.—It is now about two years since Dr. Jerry Robinson introduced his textile metallic filling material. During this time it has not only been brought to the notice of the profession pretty generally, but it has apparently won a place for itself among the lists of filling materials that is high up among the reliables.

Two years time is scarcely sufficient to judge of its enduring qualities, yet the test of this time gives great promise for the future. With many, it has taken the place of amalgam in about eighty or ninety per cent, of cases ; this alone is a good recommendation for it.

We, in common with others, have used it largely for the past couple of years and find, so far as we have yet been able to see, that it returns well for the labor of putting it in. It is not advisable to introduce it in very large pieces, as they cannot be handled with accuracy. The filling may be started with a cylinder or wad, the balance packed in, in the form of strips, such as heavy gold foil is cut into. *Thorough* malleting throughout the whole process of filling is very necessary, for, if not made dense, the filling, especially one on an approximate surface, will distintegrate under the wear of mastication. In preparing cavities on the proximate surfaces of bicuspids and molars, it is best to cut the lateral walls perfectly square, as, if they are flared, it is next to impossible to make the joints perfect, and besides this would leave a very thin portion of the material to guard the enamel.

It adds greatly to the density of these fillings to use a matrix, as the mallet can then be used liberally and without fear of dislodging any portion of the filling. The only objection so far apparent is, that when used in combination with gold and both metals exposed to the fluids of the mouth, the textile material will become black.

This is a slight fault that may be remedied to some extent by not exposing the material in places where it will be seen. Furthermore, the darkening appears to be on the exposed surface and not in the interior of the tooth. If no harm results to the tooth from the discoloration of the metal, it may pass without comment.—*Dental Register*.

---

ACID DYSPEPSIA.—In a paper read before the Manchester (England) Medical Society, Dr. McNaugh claims, from experiments made on himself, that the acid which causes the irritation in heartburn is hydrochloric acid. He analyzed matter obtained from his own stomach when he was suffering from acidity, and was thus led to the above conclusions. He further showed that the tendency of hydrochloric acid is to prevent lactic fermentation, and he adduces this as additional evidence that the acidity in acid dyspepsia is not due to lactic acid.

We are willing to concede the fact as above stated, but we repudiate the deductions. The author of the paper displays that unfamiliarity with this subject which is at the root of the empirical and often mischievous treatment of acid dyspepsia by means of alkalies, etc. This condition may be due either to an excess or a deficiency of hydrochloric acid, and the treatment differs accordingly. When hydrochloric acid is deficient, the process of normal digestion gives place to fermentation, in which lactic and butyric acids are both generated. In the case of excessive secretion of hydrochloric acid the acidity will be found to be greatest either before meals, and is relieved by food, or immediately after meals. In deficiency of this normal ingredient of the gastric juice the food remains undigested, and in from two to four hours after its indigestion, according to the nature of the food, fermentation and acidity supervene. In the latter case the eructations are not only acid but peculiarly irritating in the æsophagus. the existence of butyric acid being particularly apparent to the taste.

In the treatment of each of these varieties of acidity, or acids are to be exhibited but in an intelligent manner, and in conformity to the physiological law that acids check acid secretions. The exhibition of hydrochloric acid in combination with the simple bitter tonics one or two hours before meals overcomes to a degree the excitability of the glands, and thus renders them less susceptible to the irritation of food, the bitters assisting, by their direct tonic action on the tissue, toward permanent relief. When a deficiency of hydrochloric acid is secreted, this should be supplied immediately after each meal. The acid given at this time facilitates digestion, and thus prevents that fermentation which manifests itself in lactic and butyric acid eructations. The joint exhibition of pepsin in such cases aids in digestion.—*Medical Age.*

---

GERMS AND GERMICIDES.—J. C. Peters, M. D., writes: Carbolic acid, so-called, is not an acid, but an alcohol, and bacteria will live in alcohol for months. Nedwetsky made experiments on bacteria, in 1872, and found that they lived in strong solutions of carbolic acid; to strong to be used upon sick persons. Calomel and quinine also did not kill them, nor nux vomice; while dilute acids—nitric, surphuric, and tartaric acid, killed them, in five per cent. solutions. Tannin and gallic acid are also efficient germicides. Corrosive sublimate, one to twenty thousand parts of water, is safe and good; also iodine, one to five thousand of water; and bromine, one to fifteen hundred of water; chlorine water requires two days to kill them; a five per cent. solution of chloride of lime, ten days. Sulphurous acid, generated by burning sulphur, the most commonly used disinfectant, requires thirty minutes to kill them; and the spores in micro-cocci will often live ninety-six hours in it. A five per cent. solution of chloride of zinc requires a month to destroy them, and they have been found alive and active at the end of that time. Turpentine, which has always had an approved reputation against puerperal fever is active against bacteria, in one part to seventy thousand. The oils of peppermint and cloves are equally efficient. Benzoic acid, and benzoate of soda are inefficient.—*N. Y. Med. Planet*

